# Inhibition of endoplasmic reticulum stress alleviates triple-negative breast cancer cell viability, migration, and invasion by Syntenin/SOX4/Wnt/β-catenin pathway via regulation of heat shock protein A4

**DOI:** 10.1080/21655979.2022.2062990

**Published:** 2022-04-20

**Authors:** Jinniang Nan, Xuguang Hu, Binbin Guo, Meiyun Xu, Yufeng Yao

**Affiliations:** a School of Clinical Medicine, Nanchang Medical College, Jiangxi Province, Nanchang, P.R.China; b Department of Organ Transplantation, Jiangxi Provincial People’s Hospital, Nanchang, Jiangxi Province, P.R.China; c Department of General Surgery, Jiangsu Cancer Hospital & Jiangsu Institute of Cancer Research & the Affiliated Cancer Hospital of Nanjing Medical University, Nanchang, Jiangxi Province, P.R.China

**Keywords:** Endoplasmic reticulum stress, triple-negative breast cancer (TNBC), HSPA4, Syntenin/SOX4, Wnt/β-catenin pathway

## Abstract

Endoplasmic reticulum stress (ER stress) is a double-edged sword in the occurrence and development of malignant cancer. The aim of this study was to explore the roles of ER stress in metastasis and epithelial-mesenchymal transitionin triple-negative breast cancer (TNBC) and potential mechanisms. In this study, 4-PBA was administrated to inhibit the ER stress. Cell viability was evaluated using a cell counting kit-8 assay. Cell migration and invasion were identified by wound healing and transwell assay, respectively. Levels of MMP2 and MMP9 were measured by enzyme-linked immunosorbent assay and immunohistochemical staining. Western blot assay was used to assess the levels of ER stress-related proteins, Syndecan-1 (SDC-1)/Syntenin-1 (SDCBP-1)/SRY-related HMG-box 4 (SOX4) signaling and Wnt/β-catenin signaling. Moreover, a xenograft mice model was conducted to confirm the role of ER stress in TNBC. The data indicate that the ability of viability and metastasis of breast cancer cells were stronger than normal mammary epithelial cells. More aggressiveness was manifested in TNBC cells than that in non-TNBC cells. 4-PBA significantly suppressed the viability, migration, and invasion in BC cells and inhibited the SDC/SDCBP/SOX4 axis and Wnt/β-catenin signaling. Furthermore, heat shock protein A4 (HSPA4) overexpression stimulated ER stress and activated the SDC-1/SDCBP-1/SOX4 pathway and Wnt/β-catenin signaling. Animal experiments showed similar results that 4-PBA repressed tumor growth and inactivated the two pathways, while HSPA4 overexpression reversed the effects of 4-PBA. In summary, inhibition of ER stress inhibited TNBC viability, migration, and invasion by Syntenin/SOX4/Wnt/β-catenin pathway via regulation of HSPA4 in vivo and in vitro.

## GRAPHICAL ABSTRACT

**Figure:**
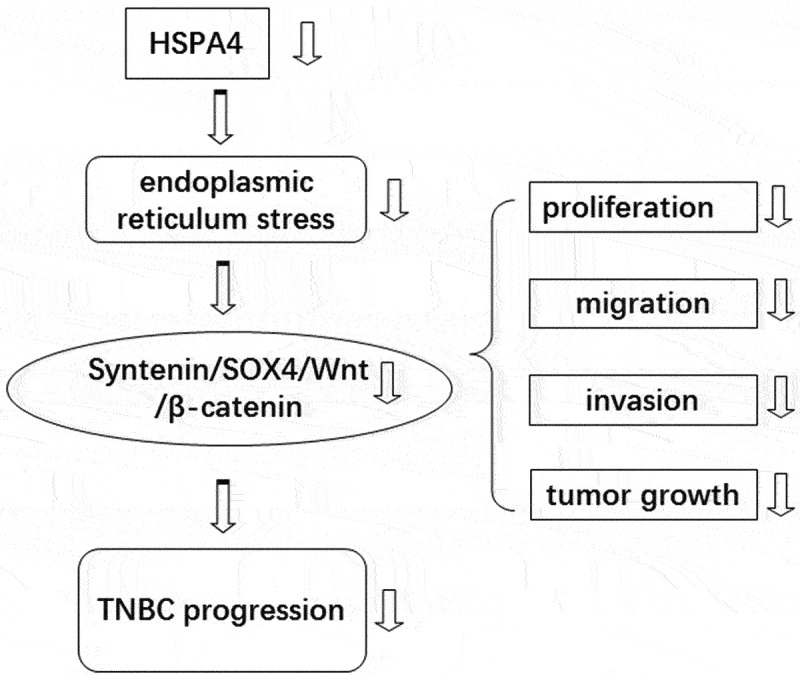

## Introduction

Triple-negative breast cancer (TNBC) is a type of breast cancer (BC) characterized by lack of expressions of estrogen receptor (ER), progesterone receptor (PR) and HER2 [1–4]. TNBC is usually more aggressive than other BC, with greater viability, migration, and invasion, metastatic activity, and lower drug sensitivity [5]. Due to the high heterogeneity of TNBC and the lack of ER, PR, and HER2 expression, it is difficult to perform standardized TNBC treatment regimens and to discover new targeted therapy [6,7]. Therefore, there is an extremely urgent need to identify potential pathogenic mechanisms and new therapeutic strategies of TNBC.

Endoplasmic reticulum stress (ER stress) plays an important role in many types of diseases, including tumors [8]. ER stress itself is an adaptive stress response to intracellular unfolded/misfolded protein [9]. In cancer cells, rapid proliferation and lack of blood supply may cause hypoxia, lack of glucose, and nutrient supply, leading to protein glycosylation and ATP synthesis [10]. These pathological changes result in a large accumulation of unfolded proteins and activate the ER stress response [11]. It is reported that ER stress promotes tumor angiogenesis by accelerating the synthesis and secretion of angiogenic factors such as angiogenin, VEGF, and PD-ECGF in the early stage of tumors [12]. Besides, the removal and reuse of unfoldable/misfolded proteins also are converted to the material base for the growth of tumor cells under an adverse environment with low oxygen and low glucose [13]. Therefore, high ER stress level in tumor tissues is usually considered to be a phenomenon beneficial to survival and proliferation [14]. However, recent studies also suggest that higher level of ER stress may lead to the tumor cell apoptosis, thereby suppressing the development of tumor [15,16].

It is well documented that the heat shock protein family can participate in the process of ERs [17,18]. Among the family, HSPA5, also known as GRP78, is the main protein that activates a series of downstream stress responses in ERs [19]. Many studies have shown that the upregulation of GRP78 can cause the activation of ERs [20–22]. In addition, heat shock protein A4 (HSPA4) is also one of the key proteins accompanying the activation of GRP78 in ERs, playing a significant role in the activation of ERs [23,24]. In this study, we aimed to explore the influence and mechanism of ER stress in TNBC. We hypothesized that ER stress is associated tightly with the functional phenotype, including viability, migration, invasion, and Wnt pathway and Syntenin/SOX4 axis. In addition, we analyzed the effects of HSPA4 on ER stress, Syntenin-1/SOX4 and Wnt pathway in TNBC in vivo and in vitro.

## Materials and methods

### Cell culture and treatment

Human non-tumorigenic breast epithelial cell line MCF-10A, triple negative breast ductal carcinoma HCC1937 and non-triple-negative breast ductal carcinoma HCC1954 were purchased from the American Type Culture Collection (ATCC, Manassas, VA) and cultured in Dulbecco’s modified Eagle’s medium (DMEM; Gibco, USA) including 10% FBS, 100 μg/mL streptomycin, and 100 U/mL penicillin in a humidified atmosphere with 5% CO_2_ and 95% air at 37°C. The inhibitor of ER stress 4-phenyl butyric acid (4-PBA; 2 or 5 mM) dissolved in PBS was added into the cells for 2 h to inhibit ER stress.

### Cell transfection

HSPA4-specific pcDNA overexpression vector (Ov-HSPA4) and corresponding control including empty pcDNA (Ov-NC) were completed by Gene Pharma (Shanghai, China). These vectors were transfected into HCC1937 cells using Lipofectamine 2000 reagent (Invitrogen, USA) according to the manufacturer’s instructions. After 48 h transfection, cells were collected for subsequent experiments.

### Cell counting kit-8 (CCK-8) assay

HCC1937 cells were transfected with HSPA40 overexpressed vectors or negative control for 48 h before treatment with 5 mM 4-PBA for 2 h. After incubation for 24 h, 48 h, and 72 h, 10 µl CCK-8 solution was added to each well at 37°C for 2 h, and then the absorbance at 450 nm was detected with a microplate reader (Bio-Rad, La Jolla, CA, USA). Six parallel wells were set up for each group, and each experiment was carried out in triplicate [25].

### Wound healing assay

Cell migration was evaluated by a wound healing assay [26]. Cells were transfected with HSPA40 overexpressed vectors or negative control for 48 h, followed by a treatment with 5 mM 4-PBA for 2 h. Then, the cells were seeded into a six-well plate and cultured to grow to 90% confluency. A 20-μl tip was used to make a straight scratch and washed three times in a serum-free medium. After 48-h incubation, the area occupied by migrated cells in the scratch was evaluated. The migration rate was calculated using the formula (area of the wound area at 0 h− the wound area at 48 h)/the wound area at 0 h.

### Transwell assay

The transwell chambers (Corning Costar, Cambridge, MA) were first coated with 0.1 mL of matrigel (Becton Dickinson, MA) at 37°C for 1 h. Cells were transfected with HSPA40 overexpressed vectors or negative control for 48 h and underwent 5 mM 4-PBA treatment for 2 h. The treated cells were collected and suspended at a final concentration of 5 × 10^5^ cells/mL in serum-free DMEM. Cell suspensions were then loaded into the upper wells, and a medium with 10% FBS was loaded in the lower chamber. After incubation for 24 h, cells on the upper surface were wiped off. The cells on the lower face were fixed with 100% methanol, stained with hematoxylin and eosin and counted under a microscope. Five randomly chosen fields were counted for each group [27].

### Quantitative real-time polymerase chain reaction (qRT-PCR)

Total RNA was extracted from transfected HCC1937 cells by Trizol reagent (Invitrogen, Carlsbad, CA, USA) in accordance with the manufacturer’s protocol. NanoDrop 2000 (Quawell, San Jose, CA, USA) was used to detect the quality and concentration of RNA, which were at 260 and 280 nm. Then, the RNA was transcribed to cDNA using the iScript cDNA Synthesis Kit (Bio-Rad Laboratories, Milan, Italy). Amplification of the cDNA was performed by real-time quantitative PCR using the SYBR Premix Ex Taq™ II kit (Takara, Shiga, Japan). The primer sequences for PCR are presented as below: HSPA4: 5ʹ- GCAGACACCAGCAGAAAATAAGG-3ʹ, 5ʹ- TCGATTGGCAGGTCCACAGT-3ʹ, GAPDH: 5ʹ-TGACTTCAACAGCGACACCCA-3ʹ, 5ʹ-CACCCTGTTGCTGTAGCCAAA-3ʹ. The HSPA4 mRNA gene was normalized to human GAPDH using the 2^−ΔΔCT^ method [28].

### Enzyme-linked immunosorbent assay (ELISA)

The contents of MMP2 and MMP9 in each group were determined with ELISA kits according to the relevant kits. The color absorbance at 450 nm was measured by a microplate reader (Winooski, VT, USA). Six parallel wells were set for each group, and each experiment was carried out in triplicate.

### Xenograft experiments

This study was approved by the Animal Care and Use Committee of The Affiliated Cancer Hospital of Nanjing Medical University (approval no. 202018527) and conducted in accordance with the guidelines established by this committee. Female Balb/c nude mice (4 weeks of age) were provided by the Institute of Laboratory Animal Science, Chinese Academy of Medical Sciences (Beijing, China). The animals were housed under controlled conditions, including a room temperature of 22 ± 1°C and alternating 12-hour light and dark cycles. The animals were fed with standard food pellets and water ad libitum. The mice were randomly divided into five groups (n = 5). The Cold Matrigel matrix was mixed at a 1:1 ratio with cell suspension in cold PBS. Mice were injected subcutaneously with 100 μl mixture (50 μl Matrigel + 50 μl cell suspension, 1 × 10^6^ cells/100 μl) on flanks. Afterward, mice were fed without or with 1 g/kg/day of 4‐PBA supplemented in the drinking water. The tumor volumes were examined twice a week by calculating the tumor volume as (length × width^2^)/2. After 3–5 weeks, mice were euthanized by cervical dislocation, and tumor tissues were then weighed and collected for histopathological examination, immunohistochemistry, and western blot assay.

### Histopathological examination (H&E) staining

Tumor tissues and lymph nodes were harvested, fixed in 10% buffered formalin and embedded in paraffin. The embedded tissues were then cut into a 4-μm thickness and stained with hematoxylin-eosin (H&E). The pathological changes and tumor metastasis were observed with a light microscope [29].

### Immunohistochemical staining

TNBC tissues fixed in 4% paraformaldehyde, dehydrated, embedded in paraffin wax, and then cut into 5 µm sections. Antigen retrieval was performed in citrate-buffered solution (pH 6.0) by microwaving for 3 min. Following blockage with 10% goat serum for 30 min, the sections were incubated overnight with Ki-67 antibody (1:300, Ab15580; Abcam) at 4°C. After that, sections were incubated with HRP-labeled goat anti-rabbit secondary antibody (1:1000, Abcam) with sustaining for 30 minutes, stained with 3, 3-diaminobenzidine tetrahydrochloride (DAB) and counterstained with hematoxylin. A light microscope (Olympus Corp., Tokyo, Japan) was used to observe the images [30].

### Western blot analysis

The total proteins from tissues or cells were extracted using RIPA buffer (Auragene, Changsha, China). Lysates were heated at 95°C for 5 min, separated on 10% SDS-PAGE (Bio-Rad, Hercules, CA) and transferred onto PVDF membranes (Millipore, USA). Next, the membrane was blocked in 5% nonfat milk for 1.5 h and incubated overnight at 4°C with the primary antibodies directed against HSPA4, HSPA5, CHOP, Syndecan-1 (SDC-1), Syntenin-1 (SDCBP-1), SOX4, FZD4, β-catenin, and GAPDH (Abcam). Membranes were washed and incubated with horseradish peroxidase-labeled secondary antibody (Cell Signaling Technology) for 1 h. The GAPDH was used as a loading control. Signals were detected using an ECL detection system (Beyotime Institute of Biotechnology, China) and quantified by densitometry (QuantityOne 4.5.0 software; Bio-Rad Inc., USA).

### Statistical analysis

All data were reported as mean ± standard deviation of three independent experiments. All statistical analyses were performed using SPSS 17.0 software. Differences among multiple groups were analyzed by one-way ANOVA followed by Bonferroni post hoc test. Values of p < 0.05 were considered to be statistically significant.

## Results

In this study, we studied the role of HSPA4 and ER stress in TNBC cell viability, migration, and invasion by Syntenin/SOX4/Wnt/β-catenin pathway. The results revealed that ER stress level and metastasis capacity were enhanced in BC cells. Suppression of ER stress inhibits metastasis, ER stress, and Syntenin/SOX4/Wnt/β-catenin pathway in BC cells. In addition, HSPA4 overexpression activated ER stress and Syntenin/SOX4/Wnt/β-catenin pathway in TNBC cells, and also stimulated TNBC tumor growth in 4-PBA-treated mice.

### Stronger metastasis capacity and higher ER stress level are observed in BC cells

**Figure 1.**
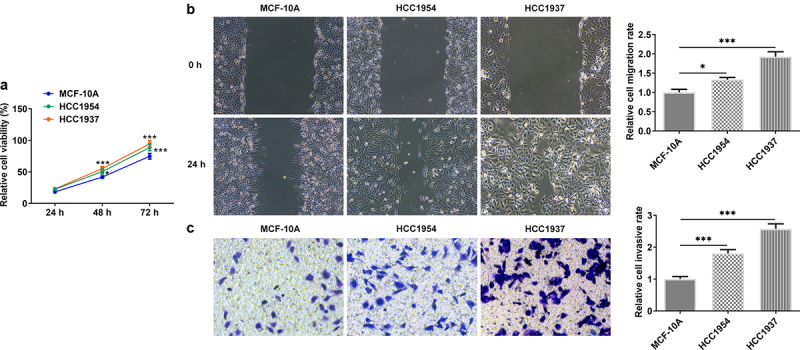
The comparison of cell viability, migration and invasion in normal mammary cells and BC cells HCC1937 and HCC1954. (a) Cell viability was detected by CCK-8 assay. (b) Wound healing assay was performed to assess cell migration. Original magnification 100 ×. (c) Cell invasion was evaluated by transwell assay. Original magnification 100 ×. Data are expressed as mean ± SD. *P < 0.05, ***P < 0.001.

**Figure 2.**
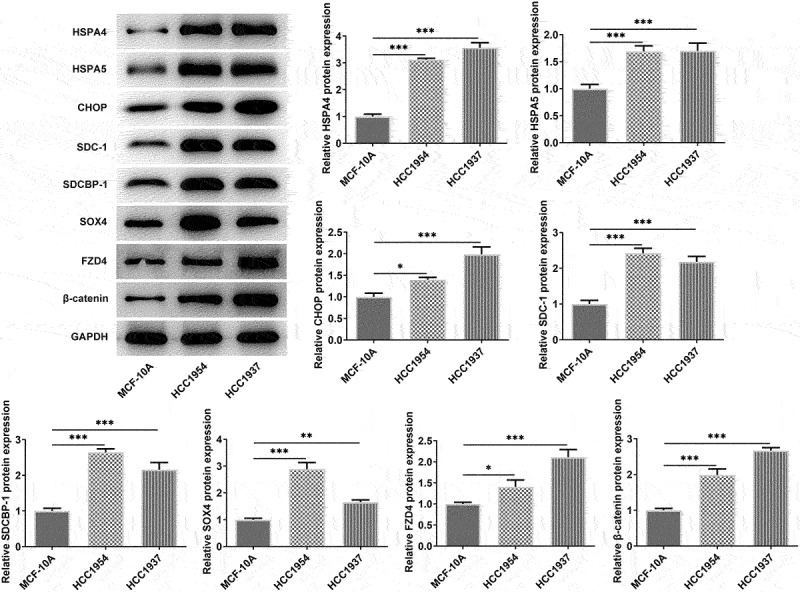
The comparison of ER stress, expressions of SDC-1/SDCBP-1/SOX4 axis and Wnt/β-catenin pathway in normal mammary cells and BC cells HCC1937 and HCC1954. Western blot assay was carried out to identify the protein expressions of HSPA4, HSPA5, CHOP, SDC-1, SDCBP-1, SOX4, FZD4 and β-catenin in MCF-10A, HCC1937 and HCC1954 cells. Data are expressed as mean ± SD. *P < 0.05, **P < 0.01, ***P < 0.001.

In this study, we first compared the differences of BC cells and normal mammary cells. As shown in Figure 1a, the cell viability was significantly increased in TNBC cell line HCC1937 and non-TNBC cell line HCC1954 compared with that in the non-tumorigenic breast epithelial cells MCF-10A. Wound healing and transwell assay also showed that the two BC cell lines had higher rate of migration and invasion when compared with the control cells (Figure 1b). Subsequently, according to results from western blot assay, the levels of ER stress-related proteins including HSPA4, HSPA5, and CHOP, compared with the control cells, were enhanced in both HCC1937 and HCC1954. In addition, BC cells showed higher contents of SDC-1/SDCBP-1/SOX4 axis and higher levels of FZD4 and β-catenin in Wnt/β-catenin pathway (Figure 2).

### Inhibition of ER stress represses metastasis, ER stress and Syntenin/SOX4/Wnt/β-catenin pathway in BC cells

**Figure 3.**
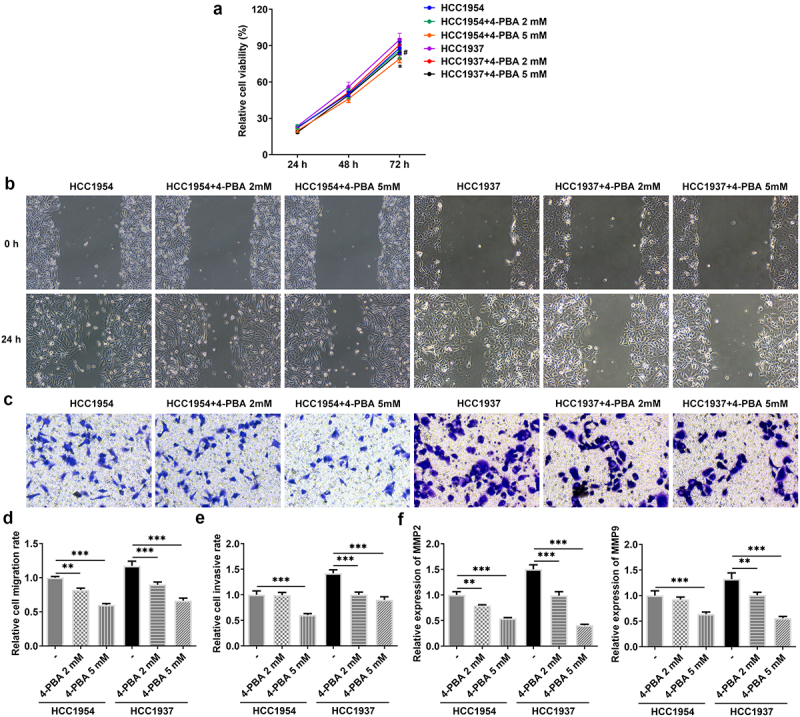
Inhibition of ER stress inhibits cell viability, migration and invasion in HCC1937 and HCC1954 cells. 2 mM or 5 mM of 4-PBA was added to inhibit the process of ER stress. (a) Cell viability was detected by CCK-8 assay. *P < 0.05 versus HCC1954. ^#^P < 0.05 versus HCC1937. (b) and (d), Wound healing assay was performed to assess cell migration. Original magnification 100 ×. (c) and (e), Cell invasion was evaluated by transwell assay. Original magnification 100 ×. (f) Western blot assay was used to detect the levels of MMP2 and MMP9. Data are expressed as mean ± SD. **P < 0.01, ***P < 0.001.

**Figure 4.**
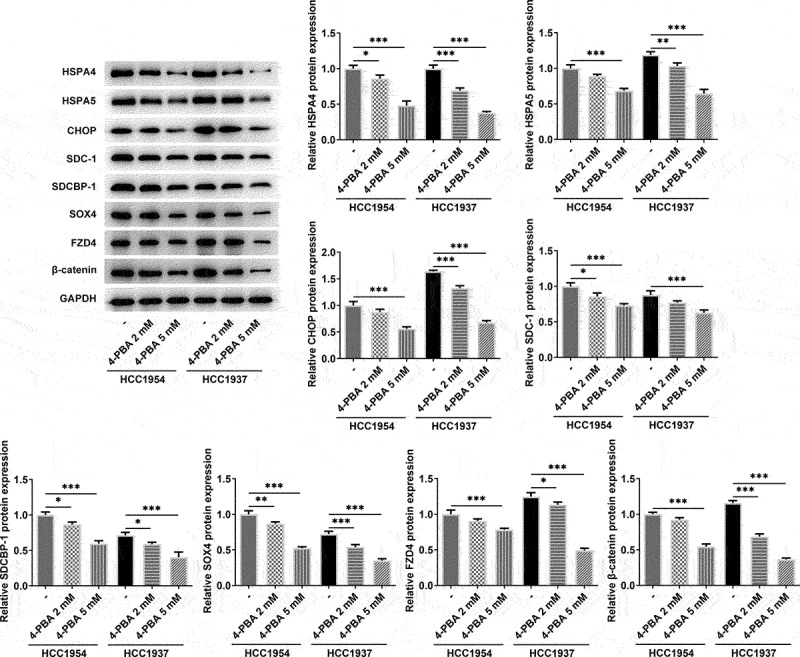
Inhibition of ER stress suppresses ER stress and Syntenin/SOX4/Wnt/β-catenin pathway in BC cells HCC1937 and HCC1954. 2 mM or 5 mM of 4-PBA was added to inhibit the process of ER stress. Western blot assay was carried out to identify the protein expressions of HSPA4, HSPA5, CHOP, SDC-1, SDCBP-1, SOX4, FZD4 and β-catenin in MCF-10A, HCC1937 and HCC1954 cells. Data are expressed as mean ± SD. *P < 0.05, **P < 0.01, ***P < 0.001.

Next, 2 mM or 5 mM of 4-PBA was used to inhibit the process of ER stress. As presented in Figure 3a, cell viability was remarkably decreased in HCC1937 and HCC1954 cells treated with 5 mM of 4-PBA compared with the untreated corresponding cell lines. Additionally, 2 mM or 5 mM of 4-PBA reduced cell migration and invasion rate in the two BC cells (Figure 3(b–e)). Consistently, the levels of MMP2 and MMP9 also were found to be decreased in BC cells after treatment with 4-PBA (Figure 3f). More importantly, the production of HSPA4, HSPA5, CHOP, SDC-1, SDCBP-1, SOX4, FZD4, and β-catenin were inhibited by addition of 4-PBA (2 mM or 5 mM) in both HCC1937 and HCC1954 cells, indicating that 4-PBA treatment suppressed ER stress and blocked the Syntenin/SOX4/Wnt/β-catenin pathway (Figure 4). Besides, Figure 4 also showed that 4-PBA had a better inhibitory effect on the signaling pathway in HCC1937, thus we chose the TNBC cell line HCC1937 for the subsequent experiments.

### HSPA4 overexpression promotes ER stress and Syntenin/SOX4/Wnt/β-catenin pathway in TNBC cells

**Figure 5.**
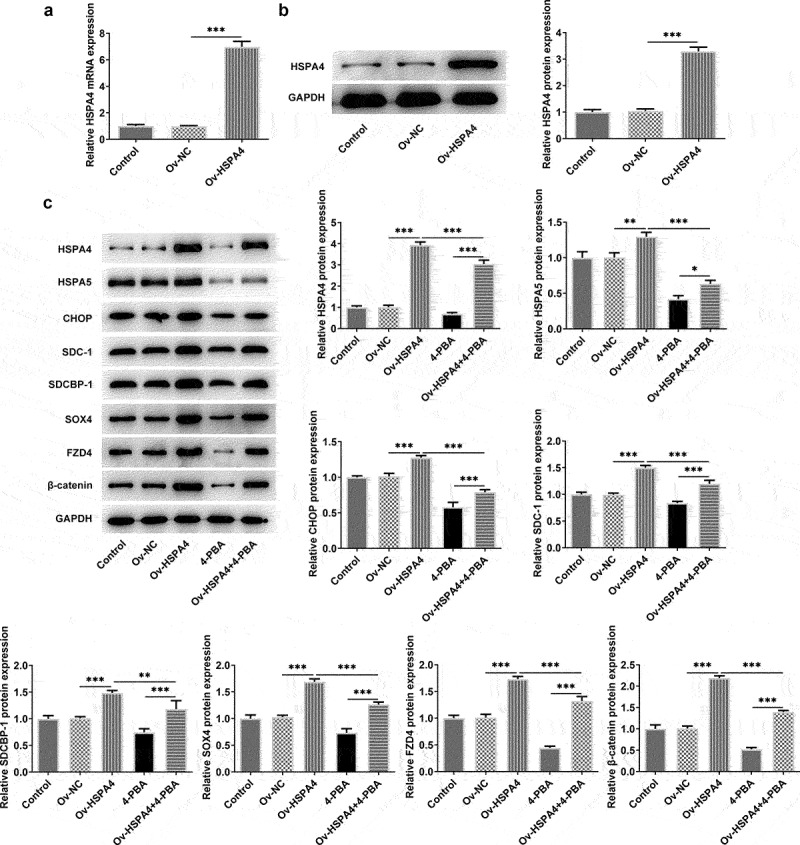
HSPA4 overexpression induced ER stress and Syntenin/SOX4/Wnt/β-catenin pathway in TNBC cells. mRNA expression (a) and protein level (b) of HSPA4 in HCC1937 were detected by qRT-PCR and western blot assay. (c) Western blot assay was carried out to identify the protein expressions of HSPA4, HSPA5, CHOP, SDC-1, SDCBP-1, SOX4, FZD4 and β-catenin in HCC1937. Data are expressed as mean ± SD. *P < 0.05, **P < 0.01, ***P < 0.001.

**Figure 6.**
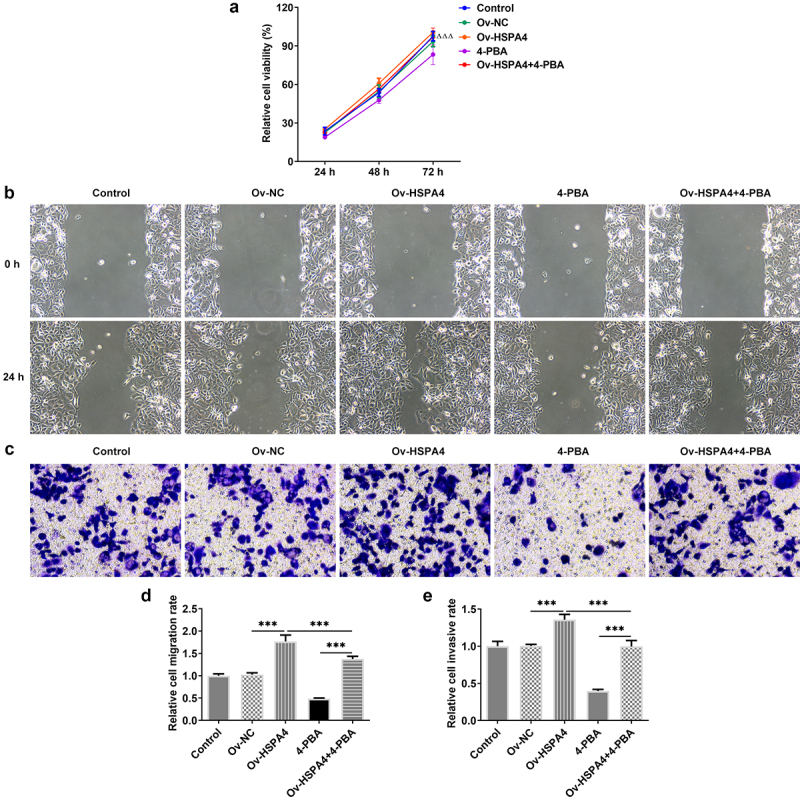
HSPA4 overexpression promotes cell viability, migration and invasion in TNBC cells. (a) Cell viability was detected by CCK-8 assay. ^ΔΔΔ^P < 0.05 versus 4-PBA. (b) and (d), Wound healing assay was performed to assess cell migration. Original magnification 100 ×. (c) and (e), Cell invasion was evaluated by transwell assay. Original magnification 100 ×. Data are expressed as mean ± SD. ***P < 0.001.

To explore the role of HSPA4 in ER stress in TNBC cells, HSPA4-overexpressed vectors were conducted and transfected into HCC1937 cells. The transfection efficiency was detected by qRT-PCR and western blot assay (Figure 5(a,b)). As illustrated in Figure 5c, HSPA4 overexpression considerably induced ER stress and increased the protein expressions of SDC-1/SDCBP-1/SOX4 pathway and Wnt/β-catenin pathway. However, co-treatment of HSPA4 overexpression and 4-PBA (5 mM) in HCC1937 cells caused the decreased levels of those proteins compared with HSPA4-overexpressed HCC1937 cells without 4-PBA treatment. CCK-8 assay results revealed that compared with the cells treated with 4-PBA (5 mM), HSPA4 overexpression significantly increased the HCC1937 cell viability (Figure 6a). Furthermore, wound healing and transwell assay showed that upregulated HSPA4 elevated the rate of migration and invasion in untreated HCC1937 cells or 4-PBA-treated cells (Figure 6(b–e)).

### Upregulation of HSPA4 promoted 4-PBA-treated TNBC tumor growth in vivo

**Figure 7.**
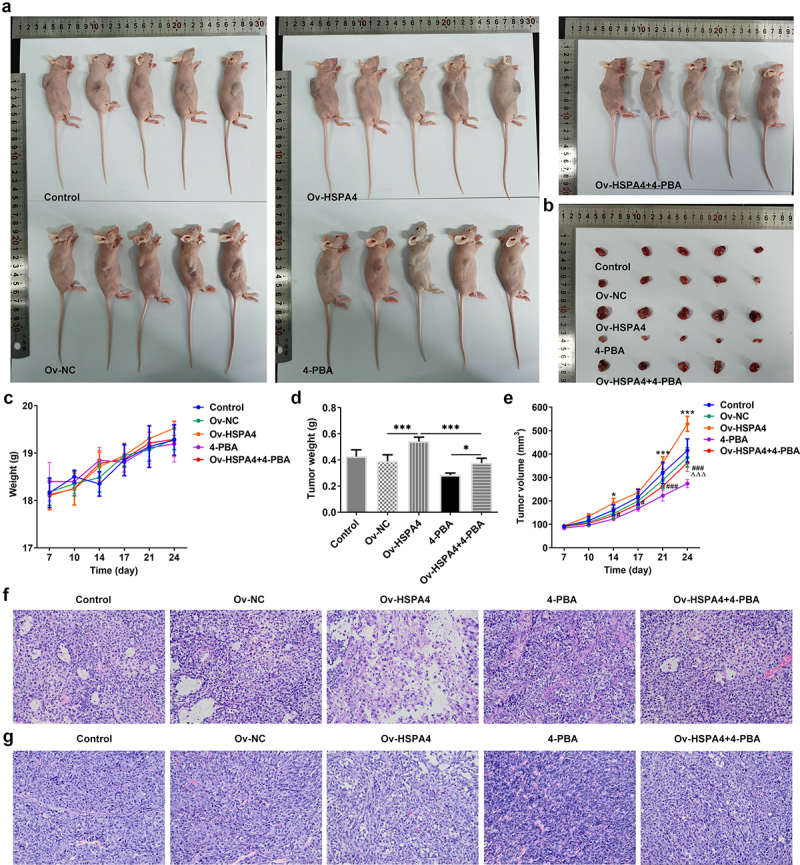
HSPA4 overexpression aggravates TNBC tumor growth in vivo. (a) (b), mice and tumor tissues transfected with or without Ov-HSPA4 in the presence or absence of 4-PBA. Body weight (c) and tumor weight (d) of mice were detected. *P < 0.05, ***P < 0.001. (e) Tumor volume was measured twice a week. *P < 0.05, ***P < 0.001 versus Ov-NC. ^#^P < 0.05, ^###^P < 0.001 versus Ov-HSPA4. ^ΔΔΔ^P < 0.001 versus 4-PBA. (f) (g), Tumor tissues and lymph nodes were observed by H&E staining. Original magnification 200 ×. Data are expressed as mean ± SD.

**Figure 8.**
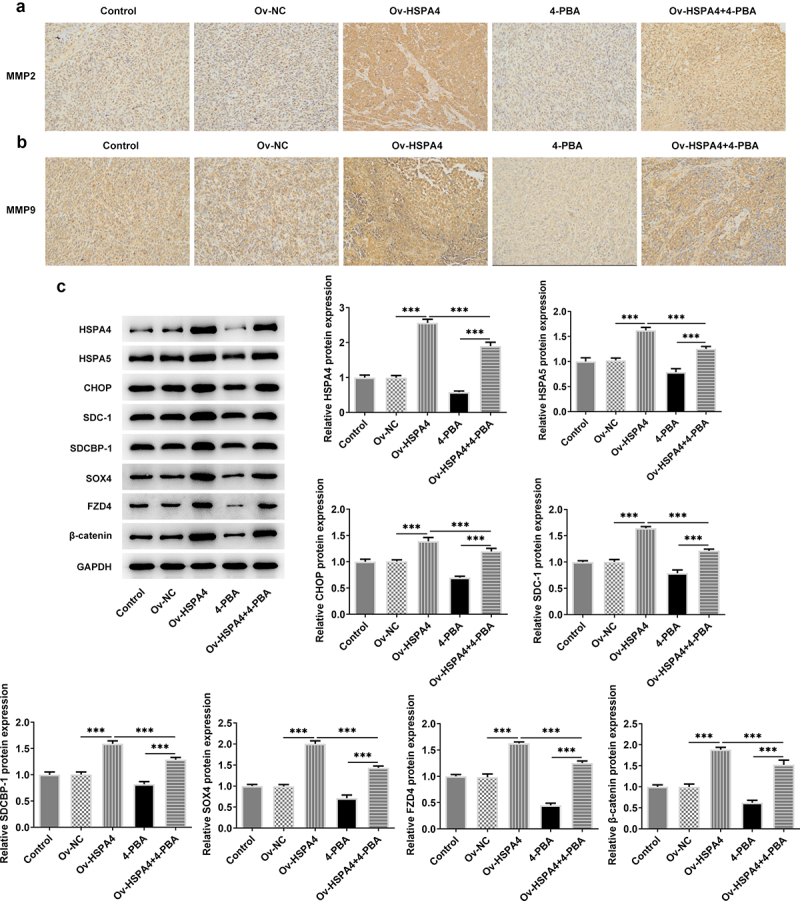
HSPA4 overexpression induces metastasis, ER stress and Syntenin/SOX4/Wnt/β-catenin pathway in TNBC mice. (a) (b), Levels of MMP2 and MMP9 were evaluated by immunohistochemical staining. Original magnification 200 ×. (c) Western blot assay was performed to identify the protein expressions of HSPA4, HSPA5, CHOP, SDC-1, SDCBP-1, SOX4, FZD4 and β-catenin in mice tissues. Data are expressed as mean ± SD. ***P < 0.001.

Finally, we established a TNBC xenograft mice model by injection with transfected HCC1937 cells (Figure 7(a–c)). The tumor weight and volume in mice are shown in Figure 6(d,e). HSPA4 overexpression greatly increased the tumor weight and volume, while 4-PBA treatment reduced them compared with the control group. However, the tumor weight and volume in mice with 4-PBA were increased after HSPA4 overexpression. As shown in Figure 7(f,g), mice in HSPA4 overexpression showed significant pathological changes, including inflammatory cell infiltration and epithelial–mesenchymal transition in tumor tissue and lymph nodes, and administration of 4-PBA reduced the pathological changes in mice transfected with Oe-HSPA4. Additionally, immunohistochemical staining showed that the levels of MMP2 and MMP9 were evidently increased in HCC1937 cells after HSPA4 was overexpressed. However, the co-treatment of HSPA4 and 4-PBA counteracted the excessive production of MMP2 and MMP9 induced by HSPA4 overexpression (Figure 8(a,b)). Expectedly, HSPA4 overexpression increased the levels of HSPA4, HSPA5, CHOP, SDC-1, SDCBP-1, SOX4, FZD4, and β-catenin in tumor tissues, but 4-PBA inhibited the generation of these proteins. The co-treatment of HSPA4 overexpression and 4-PBA showed higher levels in these proteins compared with the mice treated with 4-PBA and revealed lower levels compared with the mice transfected with Oe-HSPA4 (Figure 8c).

## Discussion

Therapeutic schedules for women presenting with TNBC are limited because of the lack of a therapeutic target and only standard chemotherapy such as paclitaxel can be employed [31]. TNBC is gradually found to be more aggressive for its stronger proliferation, metastasis activity, and lower drug sensitivity [32]. In this study, we chose three cell lines including TNBC cell line HCC1937, non-TNBC cell line HCC1954 and non-tumorigenic breast epithelial cell line MCF-10A. We compared the differences of these three cell lines in multiple aspects and found that BC cells own higher cell viability, migration, and invasion rate when compared with the control cells. What’s more, ER stress was enhanced and expression levels of the SDC/SDCBP/SOX4/Wnt pathway were significantly increased in BC cells. In addition, TNBC cells seem to be stronger in cell invasion and migration compared with the non-TNBC cells.

Syntenin, also known as MDA-9 or SDCBP, was shown as a differentially expressed gene in human melanoma cells [33]. Subsequently, it was found that Syntenin that contains the PDZ domain structurally, binds and regulates syndecans as co-receptors for various growth factors and matrix components, and regulates transmembrane receptor transport, tumor cell metastasis, and synaptic function [34–36]. Previous studies have reported that Syntenin expression was significantly increased in a variety of tumors, which promoted the invasion and metastasis of tumors [37–39]. In addition, as a binding element of SOX4, syntenin is also closely related to SOX4 [40]. Syntenin can enhance the stability of SOX4 protein and repress the degradation of SOX4 by various proteases [41]. A study has also shown that overexpression or knockdown of syntenin regulated the expression or transcriptional activity of SOX4 protein [42]. SOX4 is a common transcription factor that has been recognized to play an oncogenic role in cancers [43]. Currently, it is documented that SOX4 regulated the invasion, migration, and epithelial–mesenchymal transition (EMT) process of tumor cells through the Wnt/β-catenin pathway [44,45]. Zhang et al. have reported that the expression of SOX4 was higher in BC tissues and induced enhanced BC cell migration, invasion, and ETM [46]. In this study, ER stress in BC cells was inhibited by the administration of 4-PBA. We found 4-PBA treatment significantly suppressed the viability, migration, and invasion of BC cells. Moreover, 4-PBA reduced the levels of SDC-1, SDCBP-1, and SOX4 in the two BC cell lines, indicating that the inhibition of ER stress may block the SDC-1/SDCBP-1/SOX4 pathway, thereby inhibiting the BC progression. In addition, the inhibition of ER stress appears to have a greater effect on TNBC than non-TNBC based on the results from various functional phenotypic experiments and key protein measurements.

HSPA4 is a member of the heat shock protein family that accumulates in the cells for resistance of adverse conditions when confronting with different stresses [47]. In most cancer cells, HSPA4 is expressed abundantly to play a protective role but induces the intense ER stress [48]. Recent studies have revealed that SDC-1 as the main binding protein of Syndecan-1 can significantly decrease the expression of HSPA4 and other heat shock proteins when SDC-1 was silenced [49]. Based on this, we expected to investigate the role of HSPA4 in ER stress and the SDC-1/SDCBP-1/SOX4 pathway in BC cells. In the current study, HSPA4 overexpression reversed the inhibitory effects of 4-PBA on viability, migration, invasion, the SDC-1/SDCBP-1/SOX4 pathway and Wnt pathway in TNBC cells. Consistent with these results in vitro, animal experiments showed that HSPA4 overexpression significantly facilitated tumor growth in TNBC mice and interfered with the inhibition of 4-PBA in the SDC-1/SDCBP-1/SOX4 pathway and Wnt pathway. These results indicate that the overexpression of HSPA4 can not only further improve ER stress (to a small extent) but also promote the expressions of SDC-1/SDCBP-1/SOX4 (to a large extent), and activate the Wnt pathway (to a large extent). In addition, when 4-PBA inhibits ER stress, overexpression of HSPA4 has limited reverse effect on ER stress, but can promote the expressions of SDC-1/SDCBP-1/SOX4, while the effect on Wnt pathway is mainly caused by the upregulation of SOX4 expression caused by overexpression of HSPA4, rather than the inhibition of ER stress by 4-PBA. However, there are several limitations in this study. We only used one TNBC cell line in this work and we will verify our findings in other TNBC cell lines in case of cell line-specific effects. In addition, 4-PBA has also been reported to function as a HDAC1/2 inhibitor and HSPA4 could bind with HDAC1/2 [50,51]. The effects of 4-PBA or HSPA4 on HDACs will be analyzed to confirm that their functions in BC cells rely on ER stress. Moreover, we did not explore the clinical study to verify our results. We will provide clinical data to validate key conclusions like HSPA4 expression pattern or ER stress status in BC tumor tissues in the future. Furthermore, in this experiment, we found that HSPA4 was highly expressed in TNBC cells and overexpressed HSPA4 to explore the role of HSPA4 in ER stress or Syntenin/SOX4/Wnt/β-catenin pathway. The study in HSPA4 knockdown will further confirm our results, and we will silence HSPA4 and observe the effects of HSPA4 knockdown on ER stress or Syntenin/SOX4/Wnt/β-catenin pathway in further study.

## Conclusion

In summary, our current study revealed the relationship between ER stress functional phenotype of invasion and migration, and Syntenin/SOX4/Wnt pathway. We also demonstrated the promoting role of HSPA4 overexpression in functional phenotype and Syntenin/SOX4/Wnt pathway in TNBC cells and mice under 4-PBA-inhibited low ER stress. The inhibition of ER stress could play an important role in the regulation of TNBC, and the HSPA4-mediated Syntenin/SOX4/Wnt pathway may represent a novel therapeutic target in the treatment of TNBC patients.
